# Subchondral Trabecular Microstructure and Articular Cartilage Damage Variations Between Osteoarthritis and Osteoporotic Osteoarthritis: A Cross-sectional Cohort Study

**DOI:** 10.3389/fmed.2021.617200

**Published:** 2021-02-02

**Authors:** Feng Zhou, Linyang Chu, Xuqiang Liu, Zihao He, Xuequan Han, Mengning Yan, Xinhua Qu, Xiaofeng Li, Zhifeng Yu

**Affiliations:** ^1^Shanghai Key Laboratory of Orthopaedic Implants, Department of Orthopaedic Surgery, Shanghai Ninth People's Hospital, Shanghai Jiao Tong University School of Medicine, Shanghai, China; ^2^Department of Orthopaedic Surgery, First Affiliated Hospital of Soochow University, Suzhou, China; ^3^Department of Orthopaedics, Shanghai Jiao Tong University Affiliated Sixth People's Hospital, Shanghai, China; ^4^Department of Orthopedics, The First Affiliated Hospital of Nanchang University, The Artificial Joint Engineering and Technology Research Center of Jiangxi Province, Nanchang, China; ^5^Department of Bone and Joint Surgery, Renji Hospital, Shanghai Jiaotong University School of Medicine, Shanghai, China

**Keywords:** individual trabeculae segmentation, osteoarthritis, osteoporotic osteoarthritis, cartilage damage, subchondral trabecular boned

## Abstract

Osteoporotic osteoarthritis (OP-OA) is a specific type of OA. In this study, we aimed to assess the subchondral plate and rod microstructural differences between OA and OP-OA patients by using an individual trabeculae segmentation (ITS) system and to analyze the relationships between subchondral microstructures and cartilage damage in OA and OP-OA patients. Overall, 31 femoral heads were included in this study, which included 11 samples with OA and 13 samples with OP-OA; the normal control (NC) group contained 7 healthy femoral heads. ITS was performed to segment the subchondral trabecular bone into plate and rod trabeculae based on microcomputed tomography (micro-CT) images. We compared the plate and rod trabeculae of the subchondral trabecular bone between OA and OP-OA patients. The Osteoarthritis Research Society International (OARSI) score was employed to evaluate cartilage damage based on histological observations. Pearson's correlation coefficient and linear regression analysis were applied to analyze the relationships between subchondral microstructures and articular cartilage damage. Results showed that several microstructural parameters, including bone volume fraction (BV/TV), plate bone volume fraction (pBV/TV), rod bone volume fraction (rBV/TV), plate trabecular number (pTb.N), rod trabecular number (rTb.N), junction density between rod and plate (R-P Junc.D), and junction density between plate and plate (P-P Junc.D), were significantly decreased in patients with OP-OA compared with those in patients with OA (*p* < 0.05). Histological observations indicated that cartilage damage was more serious in patients with OP-OA than that in patients with OA (*p* < 0.05). Moreover, BV/TV, pBV/TV, pTb.N, and pTb.Th were significantly related to the OARSI score in both OA and OP-OA patients. These results indicated that there were differences in the subchondral rod and plate trabeculae between OA and OP-OA patients. Subchondral decreased plate trabeculae (pBV/TV, pTb.N, and pTb.Th) might account for cartilage damage in the progression of OP-OA. This study provided new insights to research OA when it is combined with OP.

## Introduction

Osteoarthritis (OA) and osteoporosis (OP) are two common age-related skeletal diseases that could cause disability and affect patients' quality of life (1, 2). As the most prevalent joint disorder, OA mainly causes stiffness and pain in joints (3) and is typically characterized by cartilage degeneration. The pathogenesis of OA involves entire joint including the cartilage, subchondral bone, and synovial membrane (4, 5). Interestingly, increasing evidence has demonstrated that subchondral bone turnover is an important factor in the pathogenesis of OA and is correlated with the overlying cartilage degeneration (6).

OP is a metabolic bone disease characterized by excessive bone resorption, bone microarchitecture deterioration, and fragility fractures (7). OP is frequently diagnosed in postmenopausal women (8) because estrogen decline may induce endocrinal and metabolic dysfunction, causing a predisposition to OP (9).

The relationship between OA and OP remains complicated and controversial (10). Osteoporotic OA (OP-OA) is a defined as an OA phenotype, mainly characterized by fragile and osteoporotic subchondral bones, decreased density, and high remodeling and turnover (11, 12). The sustained bone remodeling due to OP-OA might lead to subchondral microfractures, further aggravating cartilage degradation and accelerating OA progression (13, 14). In fact, experimental studies have demonstrated that cartilage damage due to OA was enhanced when OA is accompanied by OP (15). In contrast, improving the integrity of the subchondral bone could delay the progression of cartilage damage in patients with OA (16). Clinically, the deployment of anti-osteoporotic drugs for OA remains controversial; however, OP-OA patients might benefit from antiresorptive treatments (11, 17). Nonetheless, the mechanisms of how anti-osteoporotic drugs attenuate OA progression is not clear.

Previously, Liu et al. have developed the individual trabeculae segmentation (ITS) system, which is a novel three-dimensional technique for analyzing microstructural structures (18). Based on microcomputed tomography (micro-CT) or high-resolution quantitative computed tomography (HR-QCT) images, ITS could segment the subchondral trabecular bone into rod-like and plate-like trabeculae, which together make up an intricate network. By utilizing ITS analysis, more parameters about rod and plate trabeculae could be obtained, including number, density, thickness, and orientation, which is beneficial for further predicting the microarchitecture and micromechanics of the trabecular bone (19, 20). Moreover, ITS-based morphological analysis could be clinically utilized to evaluate the effectiveness of medication (21).

In our previous study (22), we compared the microstructural and biomechanical differences of subchondral trabeculae between OA and OP-OA patients using micro-CT and micro-finite element analysis. We demonstrated that microstructural changes in the subchondral bone could affect OA progression. Based on our previous study and the unique advantages of ITS, we supposed that applying ITS could further analyze the rod and plate trabecular bone between OA and OP-OA patients. By comparing the microstructural and histological differences, we hope to provide new insights to research OA when it is combined with OP.

## Materials and Methods

### Subjects

This cross-sectional study was conducted from January 2017 to December 2019 and was approved by the Ethics Committee of the Shanghai Ninth People's Hospital (No. 2018-151-T137). All patients were well-informed and provided written informed consent. Patients who had undergone total hip replacement (THA) surgery in the Department of Orthopedics, Shanghai Ninth People's Hospital were enrolled retrospectively. We excluded those who had other forms of arthritis such as rheumatoid arthritis, metabolic diseases including thyrotoxicosis, or malignancies or were taking anti-osteoporotic medications (e.g., estrogen, bisphosphonates, or selective estrogen receptor modulators) that could affect bone remodeling. Finally, 11 OA and 13 OP-OA patients were enrolled. Seven patients who had femoral neck fractures caused by high energy trauma were categorized into the normal control (NC) group; these patients did not suffer from OA or OP.

The diagnoses of OA and OP-OA were made during recruitment. OA was diagnosed based on clinical and radiographic criteria by an experienced surgeon who was blinded to the study protocol. Specifically, weight-bearing anteroposterior radiographs of the hips were obtained, and OA was subsequently graded according to the Kellgren–Lawrence (K–L) classification system by the same surgeon (23). Patients with joint pain, joint range of motion limitations that affects daily life, and K–L grade III/IV were diagnosed with OA and were enrolled to undergo THA surgery. Before THA surgery, the bone mineral density (BMD) of the lumbar spine (L1-L4) and the contralateral hip of the diseased femur was measured using dual-energy X-ray absorptiometry (DXA) (Hologic Discovery A, USA) according to a previous study (24). Participants were measured with the same densitometer at the same scan speed and were analyzed using the same software. OP was diagnosed based on an areal BMD value (*T*-score ≤ −2.5 both in the lumbar spine and femur) measured by DXA (19). Patients with OA meeting the diagnostic criteria of OP (*T*-score ≤ −2.5) were categorized into the OP-OA group.

### Micro-CT Scanning

**Figure 1:**
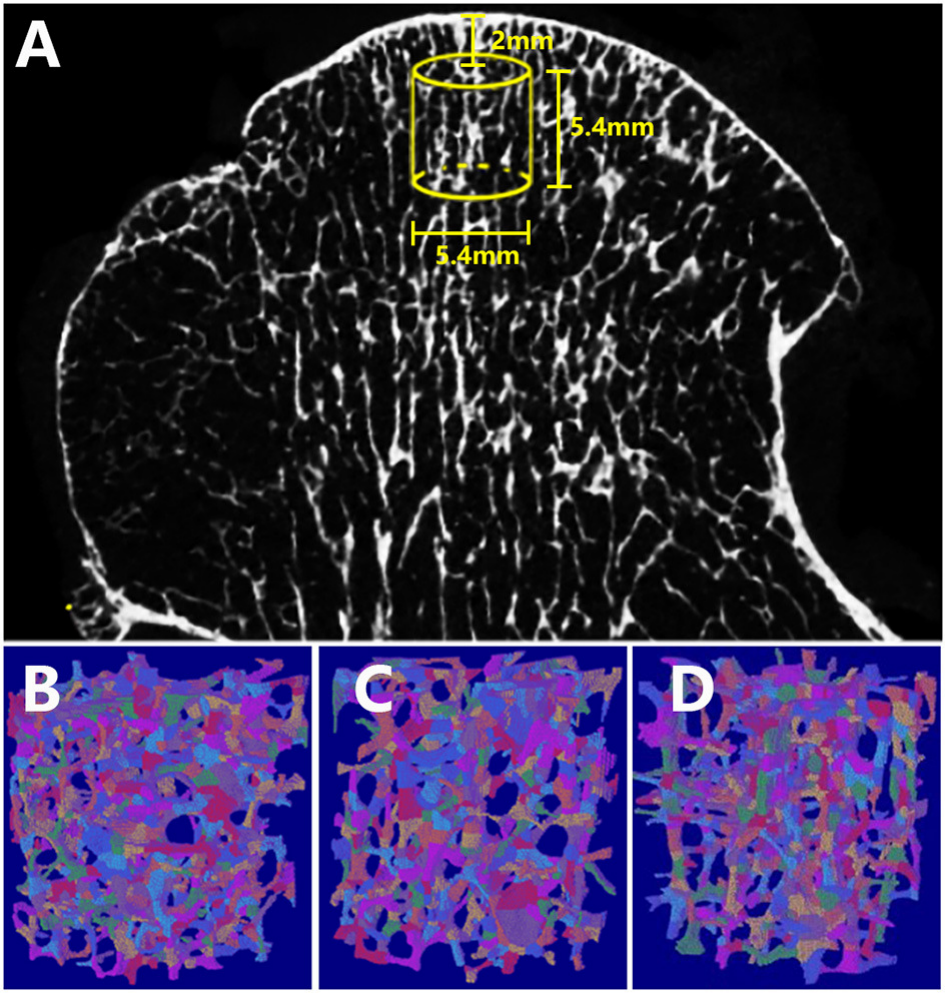
Reconstructed micro-CT and ITS images of the femoral head specimens. **(A)** The virtual cylindrical biopsies extracted from the micro-CT image (yellow cubic area indicated the volume of interest). Subchondral trabeculae of **(B)** OA, **(C)** OP-OA, and **(D)** NC groups were subjected to ITS, and were decomposed into individual rod (labeled in green) and plate (labeled in red) trabeculae.

All whole femur heads were scanned using a 70 kVp 114 μA micro-CT system (μCT 80, Scanco, Zurich, Switzerland) with a 36-μm isotropic voxel size (25). The axes of the specimens were perpendicular to the articular surface that responds to the transferred loading forces from the diseased articular surface (26). Virtual cylindrical biopsies (Ø 5.4 mm; 5.4 mm of length; 2 mm below the bone surface) were extracted as the volume of interest (VOI) in the principal stressed region using a semiautomatic contouring method. The scanned images were segmented with a low-pass filter to eliminate noise, and the bone phase was subsequently determined at the same threshold. The reconstructed three-dimensional micro-CT image was shown in Figure 1A, and the yellow cubic indicated the VOI.

### ITS-based Morphological Analysis

According to previous studies by Guo et al. (27, 28), an ITS-based morphological analysis was applied to the trabecular bone subvolumes of all specimens. The trabecular network was segmented into an individual plate and rod using complete volumetric decomposition (29). Specifically, with a digital topologic analysis, the skeletal network of the trabecular bone was transformed into a simple structure composed of surfaces and curves. Subsequently, the plate- and rod-like shapes of the subchondral microstructure were maintained. Digital topological classification was performed using a reconstruction method as previously reported; each voxel of the original image was classified as either an individual plate or rod (18). In this study, the ITS system was chosen as a primary endpoint to demonstrate microstructural differences in the OA, OP-OA, and NC groups. The ITS segmented trabeculae of various groups are shown in Figures 1B–D. Based on the analysis of separate plate or rod trabecular bones, the following ITS-based morphological parameters were evaluated: plate and rod bone volume fraction (pBV/TV and rBV/TV; %), plate and rod tissue fraction (pBV/BV and rBV/BV; %), plate and rod trabecular number (pTb.N and rTb.N; 1/mm), plate and rod trabecular thickness (pTb.Th and rTb.Th; μm), plate trabecular surface area (pTb.S; mm^2^), rod trabecular length (rTb.l; mm), junction density between rod and rod (R-R Junc.D; 1/mm^3^), junction density between rod and plate (R-P Junc.D; 1/mm^3^), and junction density between plate and plate (P-P Junc.D; 1/mm^3^).

### Histological Observation

After micro-CT scanning, the selected cylindrical VOI and the above cartilage of the specimens were decalcified in 10% ethylenediamine tetraacetic acid (EDTA) for 21 days and then embedded in paraffin. Serial sections were cut at 5 μm thickness and processed with safranin O/fast green (S&F) and hematoxylin and eosin (H&E) staining. The Osteoarthritis Research Society International (OARSI) score (grade × stage) was calculated as previously reported (30).

### Statistical Analysis

Data were expressed as mean ± standard deviation. All data were tested for normality by the Shapiro–Wilk test. An independent two-sided Student's *t*-test was used to evaluate the differences between two groups. A one-way analysis of variance was applied to compare variables among the OA, OP-OA, and NC groups. Variables were compared again after adjusting covariates by a multiple linear regression analysis. Covariates (*p* < 0.05, and independent from each other) including age, height, and weight were selected. Moreover, Pearson's correlation coefficient and linear regression analysis were applied to analyze the relationships between bone microstructure and cartilage damage. For all analyses, a two-tailed *p* < 0.05 was regarded as statistically significant. All data analyses were performed using the SPSS 22.0 statistical software package (SPSS Inc., Chicago, IL, USA).

## Results

The study population and the anthropometric data are presented in Table 1. The average age of patients with OA was 54.64 ± 13.68 years, significantly younger than the average age of 72.08 ± 7.5 years for patients with OP-OA (*p* < 0.05). The height and weight of patients with OA were 164.73 ± 7.42 cm and 64.55 ± 11.64 kg, respectively, significantly taller and heavier than patients with OP-OA (*p* < 0.05). However, there was no significant difference in the body mass index (BMI) (*p* = 0.127). The T value and BMD of patients with OA were significantly higher than those of patients with OP-OA in both the lumbar spine and femur (*p* < 0.01). Significantly different covariates including age, height, and weight were adjusted in the following comparisons.

**Table 1:** Basic information of the OA, OP-OA, and NC groups.

	**OA**	**OP-OA**	**NC**	***p*-Value** **OA vs. OP-OA**
*N*	11	13	7	–
Sex (male/female)	7/4	6/7	4/3	–
Age (years)	54.64 ± 13.68	72.08 ± 7.50	61.86 ± 22.93	0.006^**^
Height (cm)	164.73 ± 7.42	155.62 ± 7.18^b^	165.14 ± 7.13	0.005^**^
Weight (kg)	64.55 ± 11.64	53.00 ± 10.34^b^	64.86 ± 4.30	0.008^**^
BMI (kg/m^2^)	23.64 ± 2.75	21.78 ± 3.15	23.89 ± 2.59	0.127
K–L grade (K–L III/IV)	6/5	7/6	0/0	–
Femur *T-*value	−0.27 ± 1.09	−2.97 ± 0.41^b^	−0.90 ± 0.88	<0.001^**^
Lumbar *T-*value	−0.54 ± 0.97	−3.26 ± 0.57^b^	−0.66 ± 1.51	<0.001^**^
Femur BMD (g/cm^2^)	0.91 ± 0.14	0.61 ± 0.11^b^	0.80 ± 0.08	<0.001^**^
Lumbar BMD (g/cm^2^)	0.93 ± 0.10	0.69 ± 0.11^b^	0.92 ± 0.15	<0.001^**^

Symbol “*” indicates comparisons between OA and OP-OA group.

* *Indicates p < 0.05*.

** *Indicates p < 0.01*.

a *p < 0.05 between OA and NC group*.

b *p < 0.05 between OP-OA and NC group*.

Bone volume fraction (BV/TV) as a key parameter of micro-CT and ITS-based plate and rod microstructural evaluations are shown in Table 2. Generally, OA demonstrated sclerosis in the subchondral trabeculae, while OP-OA showed excessive bone resorption and microarchitecture deterioration. Specifically, BV/TV (33.16 ± 11.39%), pBV/TV (25.28 ± 7.58%) and rBV/TV (7.88 ± 4.57%) in patients with OA were, respectively higher than 22.54 ± 7.50%, 18.35 ± 6.89% and 4.19 ± 2.22% in patients with OP-OA (*p* < 0.05). Moreover, pTb.N (3.18 ± 0.27) and rTb.N (2.91 ± 0.41) in patients with OA were, respectively higher than 2.84 ± 0.36 and 2.48 ± 0.48 in patients with OP-OA (*p* < 0.05). Moreover, R-P and P-P junction densities, which indicated the connection of the trabecular network, were significantly higher in patients with OA than in patients with OP-OA (*p* < 0.05). These differences remained significant after adjustments for age, height, and weight. These results demonstrated microstructural differences of the trabecular bone between patients with OA and OP-OA.

**Table 2:** Microstructural evaluations of subchondral trabeculae and cartilage damage in the OA, OP-OA, and NC groups.

	**OA**	**OP-OA**	**NC**	***p*-Value** **OA vs. OP-OA**
BV/TV (%)	33.16 ± 11.39^a^	22.54 ± 7.50	23.32 ± 5.07	0.006^**^^#^
pBV/TV (%)	25.28 ± 7.58	18.35 ± 6.89	18.87 ± 5.63	0.021^*^^#^
rBV/TV (%)	7.88 ± 4.57^a^	4.19 ± 2.22	4.45 ± 2.05	0.010^*^^#^
pBV/BV (%)	77.56 ± 6.55	79.59 ± 12.56	80.07 ± 10.45	0.634
rBV/BV (%)	22.44 ± 6.55	20.41 ± 12.56	19.93 ± 10.45	0.643
pTb.N (1/mm)	3.18 ± 0.27^a^	2.84 ± 0.36^b^	2.47 ± 0.55	0.044^*^^#^
rTb.N (1/mm)	2.91 ± 0.41^a^	2.48 ± 0.48	2.09 ± 0.37	0.021^*^^#^
pTb.Th (mm)	0.14 ± 0.01	0.13 ± 0.01^b^	0.15 ± 0.02	0.117
rTb.Th (mm)	0.09 ± 0.01^a^	0.09 ± 0.00^b^	0.12 ± 0.03	0.940
pTb.S (mm^2^)	0.05 ± 0.00^a^	0.06 ± 0.01^b^	0.09 ± 0.03	0.627
rTb.l (mm)	0.34 ± 0.02^a^	0.33 ± 0.01^b^	0.41 ± 0.08	0.561
R-R Junc.D (1/mm^3^)	7.89 ± 4.56^a^	5.53 ± 4.23	2.99 ± 1.76	0.158
R-P Junc.D (1/mm^3^)	50.19 ± 23.33^a^	28.36 ± 16.57	16.21 ± 12.02	0.008^**^^#^
P-P Junc.D (1/mm^3^)	34.59 ± 11.08^a^	23.55 ± 11.18	14.81 ± 11.45	0.023^*^^#^
OARSI score	14.45 ± 3.95^a^	17.46 ± 3.10^b^	3.43 ± 1.62	0.030^*^^#^

Symbol “*” indicates comparisons between the OA and OP-OA groups.

* *Indicates p < 0.05*.

** *Indicates p < 0.01*.

# *Indicates that p-value remained significant after adjustment for age, height and weight*.

a *p < 0.05 between OA and NC group*.

b *p < 0.05 between OP and NC group and remained significant after adjustment for height and weight*.

**Figure 2:**
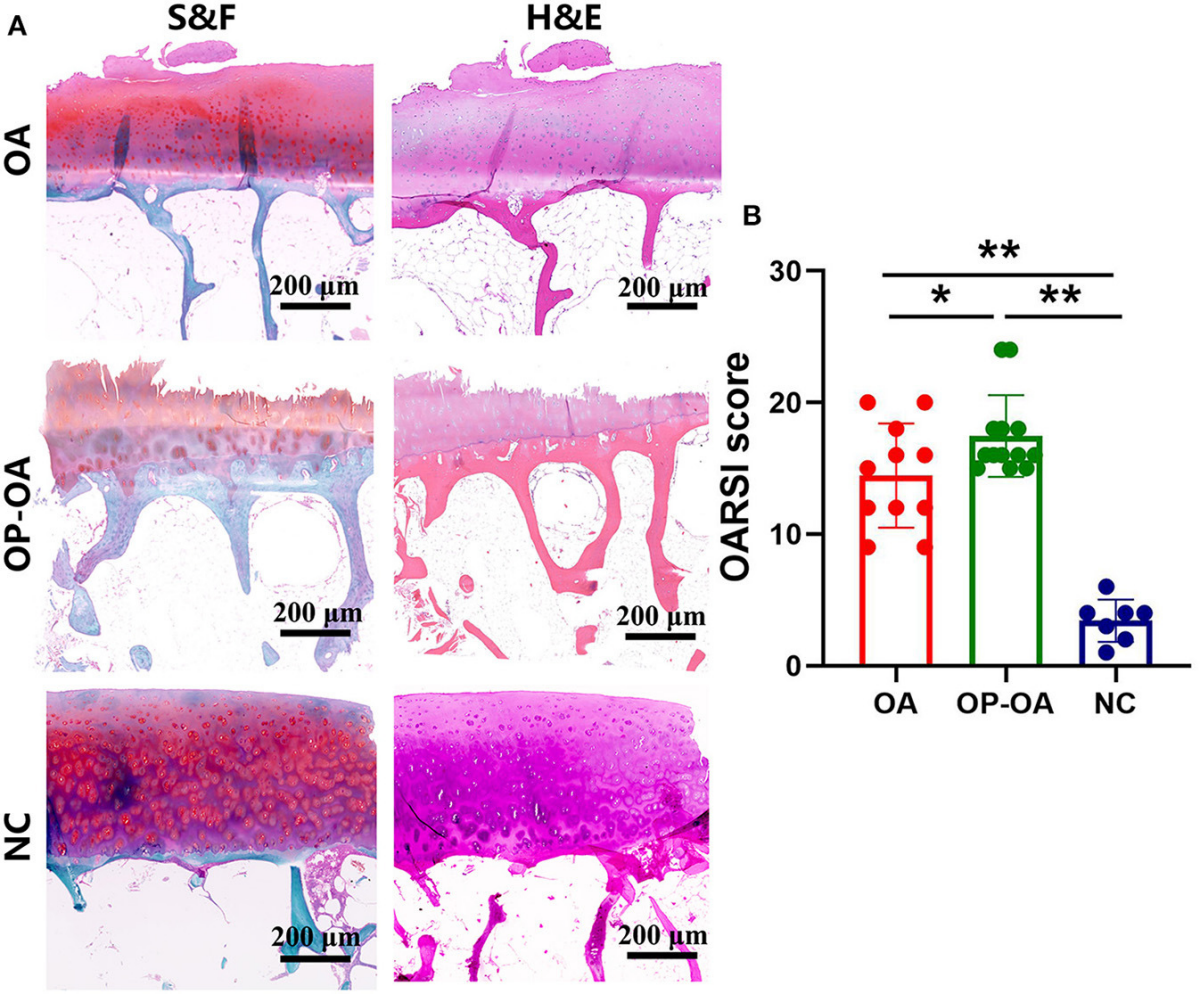
Histological observation of the articular cartilage and OARSI score. **(A)** SandF and HandE staining of the articular cartilage in OA, OP-OA, and normal patients. **(B)** The OARSI score of the cartilage in the femur head. “*” indicated *p* < 0.05; “**” indicated *p* < 0.01.

Results of histologic staining for indicating articular cartilage damage are shown in Figure 2A. S&F and H&E staining results showed that cartilage damage was more severe in patients with OP-OA than in patients with OA, as shown by increased cartilage surface destruction, proteoglycan disorders, and decreased thickness of articular cartilage in patients with OP-OA. Moreover, the OARSI score in patients with OA was significantly lower than that in patients with OP-OA, but higher than that in the control group (Figure 2B). The aggravated cartilage damage in patients with OP-OA was not caused by old age as the OARSI score in patients with OP-OA remained significantly higher than that in patients with OA after adjustment for age (Table 2).

We further evaluated the relationships between articular cartilage damage and the microstructural parameters of subchondral trabeculae using Pearson's correlation coefficient. Results demonstrated that the OARSI score was not significantly related to the microstructural parameters of the control group (*p* > 0.05); however, the OARSI score was significantly related to BV/TV, pBV/TV, pTb.N, and pTb.Th in both OA and OP-OA patients (*p* < 0.05) (Table 3). Hence, these results suggest that BV/TV, pBV/TV, pTb.N, and pTb.Th may be key microstructural factors for cartilage damage in both OA and OP-OA patients.

**Table 3:** Correlation coefficient between the microstructure and OARSI score in the OA, OP-OA, and NC groups.

	**OA**	**OP-OA**	**NC**
BV/TV (%)	−0.79^**^	−0.70^**^	−0.12
pBV/TV (%)	−0.84^**^	−0.78^**^	−0.28
rBV/TV (%)	−0.57	0.05	0.47
pBV/BV (%)	0.26	−0.77^**^	−0.43
rBV/BV (%)	−0.26	0.77^**^	0.43
pTb.N (1/mm)	−0.76^**^	−0.63^*^	−0.24
rTb.N (1/mm)	−0.72^*^	0.18	−0.04
pTb.Th (mm)	−0.72^*^	−0.65^*^	0.04
rTb.Th (mm)	0.02	−0.33	0.51
pTb.S (mm^2^)	−0.15	−0.55	0.29
rTb.l (mm)	−0.32	−0.15	0.45
RR Junc.D (1/mm^3^)	−0.55	0.29	0.16
R-P Junc.D (1/mm^3^)	−0.81^**^	−0.21	−0.11
P-P Junc.D (1/mm^3^)	−0.73^*^	−0.36	−0.12

Symbol “*” indicates correlation between the ITS-based subchondral microstructure and OARSI score.

* *Indicates p < 0.05*.

** *Indicated p < 0.01*.

**Figure 3:**
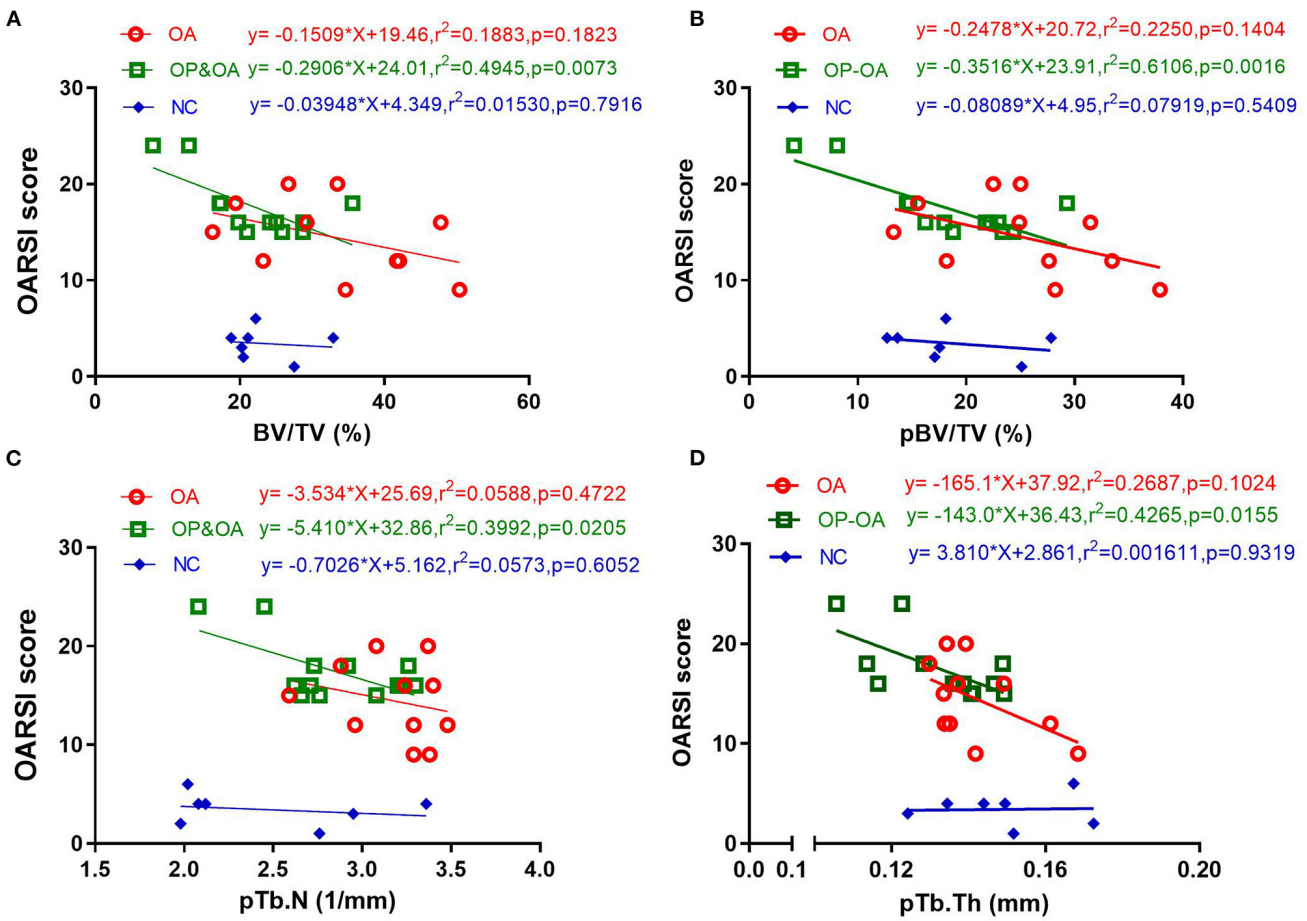
The relationship between cartilage damage and microstructural parameters of the subchondral bone. A linear regression analysis of the OARSI score with **(A)** BV/TV, **(B)** pBV/TV, **(C)** pTb.N, and **(D)** pTb/Th.

We further detected an association between articular cartilage damage and microstructural parameters using a linear regression analysis. As shown in Figure 3, in the OP-OA group, the OARSI score was significantly linearly correlated with BV/TV (*r*^2^ = 0.4954, *p* = 0.0073), pBV/TV (*r*^2^ = 0.6106, *p* = 0.0016), pTb.N (*r*^2^ = 0.3992, *p* = 0.0205), and pTb.Th (*r*^2^ = 0.4265, *p* = 0.0155). However, in the OA and control groups, the OARSI score was not linearly correlated with these parameters. These results imply that microstructural changes. especially plate trabeculae changes in the subchondral bone, may account for cartilage damage, specifically in patients with OP-OA.

## Discussion

In this study, we investigated the ITS-based microstructural analysis of subchondral trabeculae and cartilage damage in OA and OP-OA patients. Our data demonstrated that several microarchitectural parameters of subchondral trabeculae in patients with OP-OA were significantly different from those in patients with OA. Moreover, cartilage damage was more serious in patients with OP-OA than in patients with OA; this result was still valid after adjusting covariate age. Among the numerous parameters obtained from the ITS analysis, our results supported that pBV/TV, pTb.N, and pTb.Th might account for aggravated cartilage damage, especially in patients with OP-OA.

The progression of OA involves all tissues of the joint, including articular cartilage, subchondral bone, and synovium (4). As previously mentioned, the subchondral bone is a key target for OA treatment; moreover, alternating the subchondral bone is a crucial contributor to OA development. During OA progression, the subchondral bone undergoes several structural changes, which include increased bone turnover, angiogenesis, microfractures, and bone sclerosis in late stages. These changes could affect the biomechanical properties of the overlying joint cartilage (31). Increasing evidence has shown that osteoclast activity is increased in early OA stages, disturbing the equilibrium between bone formation and resorption, which could further result in a marked reduction in the subchondral bone (32, 33). Subchondral bone loss was closely associated with aggravated articular cartilage destruction (13). In advanced stages of OA, the subchondral bone was marked by increased subchondral sclerosis and bone volume (34), which may be attributed to the increased number and thickness of trabeculae (35). With the thickened subchondral plate, increased loss of aggrecan could lead to the reduced thickness of non-mineralized articular cartilage (36). In our research, specimens were collected from patients in advanced OA stages, and results showed that BV/TV, pBV/TV, and rBV/TV were increased, signifying the subchondral sclerosis and increased bone volume in OA patients.

In OP-OA, dominant subchondral bone resorption could cause inferior microstructures, thus blocking the loading stress transmission from cartilage to the subchondral bone. This further leads to increased loading stress concentrated on the cartilage layer and increased aggravated cartilage loss (22). In this study, patients with OP-OA indeed showed reduced BV/TV and aggravated cartilage damage compared with patients with OA. Moreover, this study demonstrated that plate trabeculae positively contributed to the mechanical properties of the trabecular bone. Meanwhile, plate trabeculae played a far more important role than rod trabeculae in determining the elastic moduli of the trabecular bone (19). Our results showed that patients with OP-OA had lower pBV/TV and pTb.N compared with patients with OA, while reduced plate trabeculae accounted for the inferior mechanical properties of subchondral trabeculae (24). This explained why cartilage damage was more serious in patients with OP-OA from another perspective.

Cartilage degradation may be secondary to subchondral bone alterations in OA (28). Studies have proposed that subchondral bone densification could lead to increased stiffness and further induce unbalanced stress concentration and subsequent cartilage damage (37). We demonstrated that the combination of OP might accelerate cartilage damage in patients with OA. Specifically, the structure of the cartilage surface was disrupted and the arrangement of chondrocytes was disorganized in the OP-OA group, causing a higher OARSI score. These results were consistent with a previous experimental study (15), which suggested that subchondral bone microstructure deterioration was an important factor for cartilage damage.

The ITS morphological analysis system invented by Guo et al. is a novel technique for evaluating the subchondral trabecular bone. The subchondral trabecular bone was segmented into rod-like and plate-like trabeculae, which synergistically made up an intricate network of trabecular bones (38). Based on a micro-CT analysis, ITS could further quantify the plate and rod trabeculae, thus obtaining more parameters regarding the trabeculae. Our results demonstrated that several parameters obtained from the ITS analysis, including pBV/TV and rBV/TV, were significantly different between the OA and OP-OA groups. In the control group, the OARSI score was not significantly related to microstructural parameters, while BV/TV, pBV/TV, pTb.N, and pTb.Th were significantly related to the OARSI score in the OA and OP-OA groups. Further, BV/TV, pBV/TV, pTb.N, and pTb.Th exhibited negative linear correlations with the OARSI score in the OP-OA group. These data imply that plate trabeculae might play a more important role in maintaining cartilage integrity in patients with OA.

Clinically, bisphosphonates (BPs) have traditionally been used to treat OP by inhibiting bone resorption (39). Interestingly, studies have suggested that BPs might have therapeutic effects on OA. Specifically, Neogi et al. and Fu et al. have demonstrated that the risk of joint replacement in patients with OA was lower in BP users than in non-BP users (39, 40). Theoretically, anti-osteoporotic drugs could block bone resorption and increase subchondral bone density (41). Nevertheless, advanced OA is usually accompanied by subchondral bone sclerosis; how antiresorptive drugs work on sclerotic bones remain largely unknown. It was recommended that patients with OP-OA who have elevated fracture risk should be preferably treated with BPs (11, 17). However, whether BPs should also be recommended for OA patients without OP remains unknown as randomized studies on this matter are scarce.

It is necessary to emphasize that other subchondral bone lesions may also contribute to cartilage damage. In fact, previous studies have demonstrated that the presence of subchondral bone marrow lesions was associated with deteriorated cartilage integrity in OA (42). Moreover, synovitis is another notable destructive factor that could cause cartilage degradation in the progression of OA (43). These studies confirmed that OA is a complicated disease with several pathogenic factors. In the present study, we focused on the microstructural changes of the subchondral trabecular bone, which may cause cartilage damage combined with other factors.

This study has a few limitations. First, specimens were collected from patients with advanced OA; thus, conclusions from this study might not be suitable for patients with early-stage OA. Second, CT analyses of the subchondral bone should ideally be performed *in vivo*. In fact, XtremeCT could provide a visualization of the trabecular microstructure *in vivo* (44). Third, the sample size of this study was relatively small, which may have caused selection bias. Finally, we did not detect the hormone levels that can cause bone metabolism, although we excluded patients who had a history of drugs that could affect bone metabolism. Despite these limitations, our study is valuable in evaluating the ITS-based microstructure of the subchondral trabecular bone and cartilage damage.

In summary, our study indicated microstructural differences between OA and OP-OA patients. The results imply that patients with OP-OA had aberrant subchondral trabeculae remodeling, which might have aggravated cartilage damage. Moreover, ITS-based analysis could be utilized to detect rod and plate microstructural changes in subchondral bone during OA progression.

## Data Availability Statement

The original contributions presented in the study are included in the article/Supplementary Material, further inquiries can be directed to the corresponding author/s.

## Ethics Statement

The studies involving human participants were reviewed and approved by the Ethics Committee of Shanghai Ninth People's Hospital. The patients/participants provided their written informed consent to participate in this study.

## Author Contributions

FZ, LC, XL, and ZY designed the study and drafted the manuscript. ZH, XH, and XQ analyzed the data. MY, XL, and ZY revised the manuscript for critical knowledge. All authors have read and approved the final submitted manuscript.

## Conflict of Interest

The authors declare that the research was conducted in the absence of any commercial or financial relationships that could be construed as a potential conflict of interest.
